# The value of theory in programmes to implement clinical guidelines: Insights from a retrospective mixed-methods evaluation of a programme to increase adherence to national guidelines for chronic disease in primary care

**DOI:** 10.1371/journal.pone.0174086

**Published:** 2017-03-22

**Authors:** Jessica Sheringham, Francesca Solmi, Cono Ariti, Abigail Baim-Lance, Steve Morris, Naomi J. Fulop

**Affiliations:** 1 Department of Applied Health Research, University College London, 1–19 Torrington Place, London, United Kingdom; 2 Nuffield Trust, London, United Kingdom; Escola Nacional de Saúde Pública Sergio Arauca/FIOCRUZ, UNITED STATES

## Abstract

**Background:**

Programmes have had limited success in improving guideline adherence for chronic disease. Use of theory is recommended but is often absent in programmes conducted in ‘real-world’ rather than research settings.

**Materials and methods:**

This mixed-methods study tested a retrospective theory-based approach to evaluate a ‘real-world’ programme in primary care to improve adherence to national guidelines for chronic obstructive pulmonary disease (COPD). Qualitative data, comprising analysis of documents generated throughout the programme (n>300), in-depth interviews with planners (clinicians, managers and improvement experts involved in devising, planning, and implementing the programme, n = 14) and providers (practice clinicians, n = 14) were used to construct programme theories, experiences of implementation and contextual factors influencing care. Quantitative analyses comprised controlled before-and-after analyses to test ‘early’ and evolved’ programme theories with comparators grounded in each theory. ‘Early’ theory predicted the programme would reduce emergency hospital admissions (EHA). It was tested using national analysis of standardized borough-level EHA rates between programme and comparator boroughs. ‘Evolved’ theory predicted practices with higher programme participation would increase guideline adherence and reduce EHA and costs. It was tested using a difference-in-differences analysis with linked primary and secondary care data to compare changes in diagnosis, management, EHA and costs, over time and by programme participation.

**Results:**

Contrary to programme planners’ predictions in ‘early’ and ‘evolved’ programme theories, admissions did not change following the programme. However, consistent with ‘evolved’ theory, higher guideline adoption occurred in practices with greater programme participation.

**Conclusions:**

Retrospectively constructing theories based on the ideas of programme planners can enable evaluators to address some limitations encountered when evaluating programmes without a theoretical base. Prospectively articulating theory aided by existing models and mid-range implementation theories may strengthen guideline adoption efforts by prompting planners to scrutinise implementation methods. Benefits of deriving programme theory, with or without the aid of mid-range implementation theories, however, may be limited when the evidence underpinning guidelines is flawed.

## Introduction

It is widely recognised that adoption of national guidelines remains variable and guideline adherence programmes have had limited success in improving it.[1–3] There has recently been “a wave of optimism in implementation science” that applying theory will lead to programmes with greater chance of success.[4] Similarly, there is a strong drive for theory-driven evaluation of approaches to get evidence into practice, to the extent that describing programme theory is required for peer-reviewed publication of quality improvement projects.[5] In practice, however, many ‘real-world’ programmes (i.e. where guidelines are implemented outside a research context) rarely develop programme theory prospectively.[6, 7] Opportunities to capture learning from such initiatives are limited, partly because they may not meet standards for peer-reviewed publication without articulated theory in either the programme or its evaluation. Moreover, in many apparent theory-driven evaluations, theory has not in fact shaped evaluation questions, informed methods or interpretation in any visible way.[8] Therefore questions remain about whether and how a theory-driven approach can productively influence evaluation and/or implementation initiatives in real-world circumstances.

In this paper we describe a retrospective evaluation of a ‘real-world’ guideline implementation programme using a theory-driven approach. We then use this case to provide generalizable learning and reflections on the feasibility and value of theory construction in evaluating real-world, guideline adherence programmes and how theory construction may, or may not, lead to greater programme success.

## Materials and methods

### Objectives

In this evaluation, we sought to:

Construct retrospectively the programme theory/theories used by those involved in programme delivery before and during the programme
a) Test whether the impacts predicted by theory/theories occurred using quantitative and qualitative data
  b) Derive candidate explanations for impacts or lack of them, considering the extent to which they to support or highlight flaws in the programme theory/ theories.

### Setting: The ‘Year in the Life’ Programme

The ‘Year in the Life’ (YiL) Programme sought to increase implementation of national guidance for chronic obstructive pulmonary disease (COPD) care. It started in 2010 shortly after publication of updated National Institute for Health and Care Excellence (NICE) COPD guidelines and a national strategy for COPD.[9, 10] YiL took place across four boroughs in north–east London—Redbridge, Barking & Dagenham, Havering, and Waltham Forest. The boroughs comprise 189 general practices serving a socio-demographically diverse population of approximately 1 million. Havering, which borders the county of Essex, has a predominantly older and White population. The other boroughs bordering inner London have younger and more ethnically diverse populations. The populations of Waltham Forest and Barking and Dagenham are socioeconomically disadvantaged compared with the more affluent populations in Redbridge and Havering.

A collaboration across the National Health Service (NHS), academia, and the information technology industry developed YiL with local general practitioner (GP) representatives. Measurement and monitoring of practices’ COPD care using the area’s informatics system was a core part of YiL. Planners (those involved in devising, planning, and implementing YiL, comprising respiratory clinicians, GPs, change management experts, local NHS leaders) examined practices’ baseline performance on processes of care recommended in national guidelines at the start of YiL, developed benchmarked reports on selected care processes at intervals during the programme and implemented a template in practices to standardise data recording. Alongside this, planners developed educational activities comprising masterclasses, spirometry training and nurse mentorship, and developed and distributed a self-management plan leaflet to provide practices with information to support patients with ‘rescue packs’ (antibiotics and/or steroids to keep at home in case of exacerbations). Planners sought ongoing contact with practices throughout the programme, by attending practice meetings, organising events and sending email updates.

Official programme launches were held in December 2010 and April 2011, but most activities did not start until September 2011. No official end date was given but activities were completed by December 2012.

### Design

**Fig 1:**
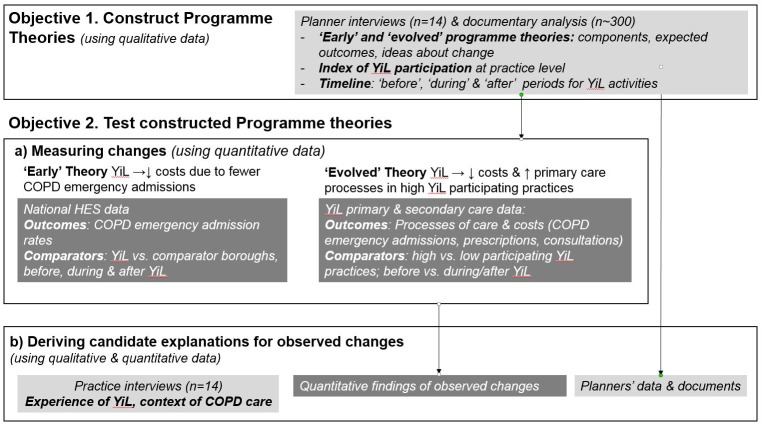
Mixed methods design to address evaluation objectives.

This study was started after the completion of all YiL activities. We combined a range of qualitative and quantitative methods to address the study’s objectives.(Fig 1)

### Objective 1. Constructing early and evolved programme theories using qualitative data

We sought to articulate the implicit theories of those close to the programme ex ante (i.e. before or during programme implementation). To do this, we built an understanding of YiL through analysis of documents (COPD guidelines and strategy, YiL reports, meeting agendas, minutes, emails and related attachments from 2010 to 2013, n~300) and semi-structured interviews with all the YiL planners and an expert in respiratory care programmes who worked across London (n = 14). An information sheet was provided to all interviewees and written informed consent was obtained prior to interview. The interviews covered planners’ recollections of the programme’s origins and development, possible programme impacts and how they thought such impacts would be achieved before, during and after the programme.

We applied framework analysis[11] with an initial coding framework based on the Template for Intervention Description and Replication (TIDieR) checklist[12] and interim YiL evaluations.[13] The TIDieR checklist prompts description of overall programme rationale; identification of individual components, who delivered them and at what intensity; what was tailored or modified and the fidelity of implementation adherence to initial plans. This checklist provided the basis for overarching codes, and analysis of documents and interviews were used to generate subcodes within them (e.g. within the code of rationale, we identified subcodes for: implementing guidelines; increasing the value of care; the power of IT and data; patient-centred care; competition between practices; reducing admissions). We described programme theory in terms of programme components, aims and mechanisms by which planners thought change would be achieved. We also constructed a timeline of implementation[14], major concurrent contextual changes, and an index of practice participation based on records of COPD template installation on practice systems, training attendance and nurse mentorship participation.

### Objective 2. Testing constructed programme theories

#### a. Measuring change using quantitative data

We conducted two controlled before-and-after analyses at national and local levels with the selection of comparator populations (local authorities matched in terms of population size, age, gender and COPD prevalence) and outcomes informed by the two constructed programme theories (described in detail in Results).

The first was a national comparison of emergency hospital admission rates between YiL and similar boroughs before, during and after the programme (testing hypotheses based on Theory 1)

Theory 1 meant the YiL evaluation ought to focus only on the outcome of emergency hospital admissions (EHA). To do this, we constructed age and sex standardised rates of COPD EHA using age and gender stratified England population (2004–2012) at borough level as the denominator.[15] The numerator comprised all patients who had an EHA for COPD (ICD 10 codes of 'J41', 'J42', 'J43', 'J44', 'J47') in Hospital Episode Statistics data (in comparator boroughs, n = 75,629; in YiL boroughs, n = 18,238 (2004–2013).

To calculate differences in COPD EHA between YIL and comparator boroughs we constructed a Poisson model with the number of admissions as the outcome variable and the log of the population included as an offset variable. We defined programme impact as the interaction of the exposure variable (YiL borough yes/no) with the analysis period variable (before/during/after YiL), after adjustment for age, gender, deprivation and month (seasonality). To ensure p-values were valid, we calculated empirical standard errors using General Estimating Equations with an independent working correlation matrix to account for the correlation of outcomes within a borough.

The second was a local comparison of processes of care, outcomes and costs between high and low participating practices before, during and after the programme (testing hypotheses based on Theory 2)

Theory 2 required a consideration of processes and outcomes. Therefore a more detailed analysis than Theory 1 was needed. We linked GP records and Secondary Users Service data from the four programme boroughs as a panel dataset (n = 513,000 patient-month observations, 2010–2014). We employed a difference-in-differences regression analysis to compare changes in outcomes among practices with moderate-to-high participation in YiL (scores 2–4) against those practices with little-or-no participation (scores 0–1) using a two-stage estimation approach. In the first stage we derived the predicted probability of each YiL outcome (as determined by theory 2) each month in the “before” period from the coefficients of multivariate population average logit (for binary outcomes) or negative binomial (for count variables) regression models with robust standard error. Subsequently, we collapsed the dataset of actual and predicted values of each outcome by practice and quarter to derive the proportions (actual and expected) of these outcomes. Finally, we examined the difference between the actual and predicted outcome (i.e. dependent variable) using regression analysis with an interaction term indicating a) whether the practice had moderate-to-high participation in YiL and b) the quarter was in the “during or after” periods of the study. In the final stage, we included fixed effects for practice and quarter.

The programme’s impact on NHS costs was evaluated by estimating the costs of programme interventions and changes in healthcare usage (primary care consultations, EHA for COPD and prescriptions).

Further details of these methods are in supplementary material (S1 Supporting Information) and in the protocol summary.[16]

#### b. Deriving candidate explanations for observed changes ex post (or lack thereof)

Others have used qualitative research methods ex post (i.e. post-implementation) to further develop or refine programme theories.[17] We have used such methods to explain observed impact ex post.

We conducted in-depth interviews with practice staff (providers) to capture experiences of YiL and the context of delivering COPD care. We used a purposive sampling approach, interviewing providers nominated by the practice (e.g. practice nurse, GPs or practice manager) who could best speak to the themes to be covered in two practices from each borough, representing practices with both high and low “uptake” of YiL component interventions. Practices were initially approached by study’s GP leads or individuals working with practices in each Clinical Commissioning Group, then followed up by ABL. An information sheet was provided to all interviewees and written informed consent was obtained prior to interview.

We used a thematic approach[18] to analyse these data together with planners’ interviews, starting with the initial coding framework developed from planners’ interviews which we adapted through codes identified inductively in the provider transcripts. Informed by Fetters et al[19], we then compared and combined all the qualitative and quantitative data to identify factors that could explain findings and/or examine whether observed changes could credibly be attributed to YiL.

### Ethics approval statement

NHS ethics approval was not required because the study did not access patient identifiable data and interviewees were all healthcare professionals. UCL ethics approval (ref: 2037/002) and NHS R&D approvals were sought and granted (ref: 148797). An information sheet was provided to all interviewees and written informed consent was obtained prior to interview.

## Results

We first describe programme theories identified from planners’ ideas ex ante and their iteration over time, focusing on three core elements of theory: programme components, expected outcomes and ideas about mechanisms for change. After setting out two testable theories derived from these ideas, we report findings of each quantitative analyses. Finally, we discuss the evidence drawn from all the data for three possible candidate explanations for observed effects post-implementation.

### Identified programme theories

Planners did not explicitly articulate programme theory during YiL. However, various implicit theories could be derived from the descriptions of programme components, expected outcomes and planners’ ideas about change in interviews and documents.

Planners overwhelmingly conveyed in their interviews that YiL developed iteratively. It was not possible to track evolution of a single theory but we condensed the various ideas about YiL into two programme theories, ‘early’ and ‘evolved’, to illustrate which ideas about the programme and its processes of implementation changed and which aspects were retained. The ‘early’ theory (early 2010 to ~late-2012) captures ideas more dominant when YiL was in the planning stages and whilst the programme was still active. The ‘evolved’ theory (~mid 2012 to late 2014) overlaps in time with the ‘early’ period to capture ideas present when YiL was in its later stages and still active and reflections after the programme.

#### Characteristics of ‘early’ theory (early 2010-end 2012)

In its early stages, YiL’s scope was fluid, with several different initiatives considered or tried, and planners described many broad goals. However, even from early in the programme, planners held some central, related ideas about what drives improvement, encompassing the power of the local informatics “solution”, the utility of clinical data and the role of clinical education in improving knowledge, and, in turn, clinical practice:

*GP level and Practice level feedback will be regularly supplied as the basis for effective population management…to help generate effective service and performance improvements during the course of the year of the project*. (YiL Leaflet, 1/2011)

There were aspirations for the programme to be an exemplar of the benefits of the IT system. However, there was little consideration of how data sharing would work, just an assumption that identifying priorities and sharing data would be sufficient stimulus for change:

*By co-producing the priority areas and actions for implementation*, *diffusion of best practice will occur across the 200 GP practices*. (YiL leaflet 2011)

Some (though not all) planners sought to use the programme as a model for using ‘value’ (which was understood by planners as reducing costs whilst improving quality) to drive improvement in other long-term conditions. We infer that planners expected costs would reduce through reducing COPD EHA but the mechanisms by which YiL was expected to achieve cost savings were unclear:

*YiL aims to demonstrate that cost and quality can be improved together at scale in the NHS*.(Board report 3/2011).

*We spend a lot of money by giving not terribly good care to people where they keep falling through the cracks and they need to be rescued in hospital*. (Planner 14, change management expert)

…*cost savings for the system (e*.*g*., *by reducing avoidable COPD admissions through more timely and proactive care in the community)*. (GP/commissioner-directed leaflet, 3/2011).

#### Characteristics of ‘evolved’ theory (mid 2012-end 2014)

Contemporary programme documentation indicated that during the programme, planners adjusted and elaborated initial ideas primarily around implementation and scope, dropping certain activities and strengthening and modifying others. This was partly due to many technical difficulties with the informatics system (as reported in an interim evaluation)[20] after initial optimism about its potential. Planners recognised problems with data quality and that data sharing alone would not stimulate improvement but their approach depended on using data sharing and feedback to drive implementation. As a result, interventions were modified to incorporate data recording elements into the COPD template, mentorship, and masterclass content. There were some tensions with the direction the programme took in its focus on data. For example, one planner critiqued the programme for fixating on supporting data capture at the expense of improving the quality of care.

In contrast to ‘early’ theory, YiL planners explicitly considered practices’ active participation in YiL as a determinant of programme effectiveness. They reflected in interviews conducted after YiL had finished that they hadn’t expected practices to require training but that many practices did not take part in the educational activities or were hard to engage in any aspect of the programme.

*We were just going to give GPs the data and the GPs would organise themselves to work on that data* (Planner 1, clinician).

Some of the practices in some of the PCT areas adopted this more readily than others. (Planner 10, manager)

In the evolved theory, planners observed they developed more focus on specific parts of guideline implementation, e.g. prioritising the quality of diagnosis:

*It came as news to all of us that we needed an additional intervention which was on this very fundamental piece about do these people have COPD or not*. *(*Planner 14, change management expert)

We *identified a huge learning need around accurate diagnosis*. (Planner 1, clinician).

Consistent with ‘early’ theory, planners still aspired to reduce costs through reducing healthcare use. The importance and feasibility of this aim was maintained by some planners even after the programme had finished.

*If it turned out not to be I would not necessarily conclude that the concept was wrong*, *it was just that we hadn’t gone about showing it in the right way*. *This was so self-evident—common sense—that if we simply do some things well and in a timely fashion*, *we can save people a lot of suffering unnecessary intervention*. (Planner 14, change management expert)

However, the mechanisms by which costs and healthcare use could be reduced were still rarely discussed in interviews or later documentation but there is some reference in the programme’s interim economic evaluation to the NICE guideline implementation report which considered ensuing reductions in admissions and primary care activity as “*likely*”.[21]

### Testable theories, timeline and major contextual changes

**Fig 2:**
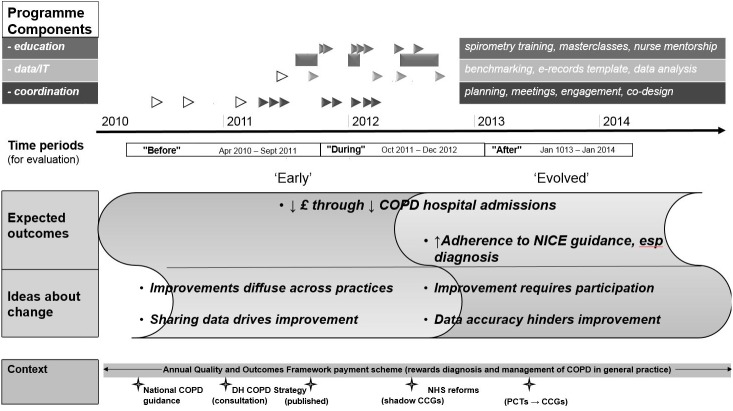
YiL implementation timeline, evaluation periods and programme theories (early and evolved). Triangles = programme events or blocks of activity (hollow = preparatory, solid = during the programme), crosses = contextual events.

The two testable theories from the ‘early’ and ‘evolved’ ideas are described below and shown on a schematic timeline, together with evaluation periods and major contextual changes.(Fig 2)

### 2a. Testing the hypotheses supporting the theories using quantitative analysis

#### Early theory

‘Early’ theory predicts that YiL would result in greater reductions in EHA in YiL boroughs than in other comparable boroughs over the same timescale. These reductions would lead to cost savings.

There was no evidence that YiL reduced emergency admissions more than comparator areas. Rates were not significantly different in YiL boroughs than comparators before, during or after the programme (Table 1). Results were similar in all alternative analyses. (Table A and Table B in S1 Supporting information)

**Table 1:** Rate ratios and 95% confidence intervals for YiL boroughs versus comparators in each time period.

Time period	Exposure	Rate ratio*	95% CI
**Before**: April 2010—September 2011	Comparator	1.000	
	YiL boroughs	1.135	(0.796, 1.619)
**During**: October 2011 –December 2012	Comparator	1.000	
	YiL boroughs	1.223	(0.839, 1.783)
**After**: January 2013 –January 2014	Comparator	1.000	
	YiL boroughs	1.183	(0.810, 1.727)

*Rate ratio = adjusted rate of emergency admissions for COPD per month per 1,000 populations in the YIL boroughs compared to the rate of admissions in the comparator boroughs. Rated were adjusted for age, gender, month (seasonality) and deprivation.

#### Evolved theory

‘Evolved’ theory predicts effects would be greater where there was greater practice participation in YiL activities. It extends ‘early’ theory by specifying outcomes of implementing the aspects of guidelines that YiL prioritised, i.e.:

- improving the quality of diagnosis by confirmation with the use of **post-bronchodilator spirometry**
- encouraging use of rescue packs by distributing a **self-management plan** leaflet
- increasing **pulmonary rehabilitation** referrals for patients with severe COPD
- reducing **prescribing of inhaled corticosteroids** without other medications.

In common with ‘early theory, it predicts that YiL would reduce EHA and thus reduce associated costs.

**Fig 3:**
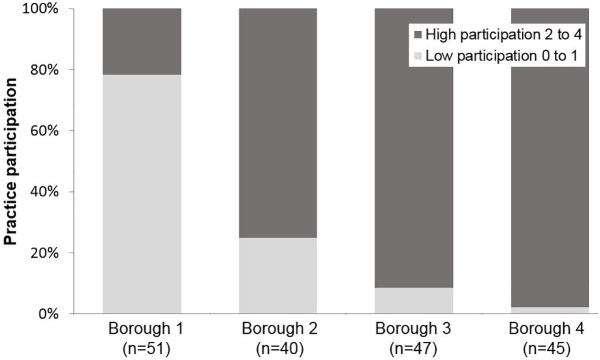
Practice participation in YiL: distribution of scores by borough. Number of interventions each practice undertook. Measured by: evidence of attendance by practice staff at YiL educational events (Spirometry training, Nurse Mentorship programme, Master Classes) or whether e-template for COPD was uploaded on practice computer.

YiL Participation by practice: Overall, 15/183 (8%) practices participated in all possible educational activities and had the template installed, with 30% having medium-high participation and significant variation by borough (Fig 3).

Adherence to NICE guidelines COPD diagnosis and management: Greater participation in YiL was associated with greater improvements in appropriate diagnosis and management advised by the guidelines, as measured by recorded post-bronchodilator spirometry, provision of a self-management plan and pulmonary rehabilitation referral.(Table 2)

**Table 2:** Difference in differences results comparing ‘before and during’ vs ‘after’ periods of YiL for practices that scored 2–4 vs those who scored 0–1.

Outcome (‘before’ vs ‘during & after’)	Before	During + after			Unadjusted difference in differences	Difference in differences, coeff (95%CI)
	Inv. 0–1, Mean (SD)(N = 330)	Inv. 2–4, Mean (SD)(N = 766)	Inv. 0–1, Mean (SD)(N = 495)	Inv. 2–4, Mean (SD)(N = 1143)		
**Diagnosis**						
*PBD spirometry (N = 2*,*734)*	0.04(0.09)	0.07(0.15)	0.18(0.18)	0.28(0.22)	0.070	0.06(0.049;0.079)**
**Management**						
*Self-management plan(N = 2*,*734)*	0.02(0.06)	0.04(0.13)	0.12(0.19)	0.20(0.23)	0.060	0.06(0.042;0.078)**
*Pulmonary Rehab (ever) (N = 2*,*728)*	0.006(0.02)	0.03(0.07)	0.02(0.06)	0.08(0.13)	0.036	0.04(0.026;0.046)**
*Inhaled cortico-steroid prescribing (N = 2*,*734)*	0.004(0.012)	0.004(0.012)	0.002(0.008)	0.0017(0.007)	-0.001	-0.001(-.003;0.0003)
**Health care use**						
*COPD emergency admissions (N = 2*,*734)*	0.007(0.01)	0.008(0.01)	0.008(0.01)	0.009(0.01)	0.000	-0.0001(-0.002;0.001)
*GP visits (N = 2*,*734)*	0.66(0.25)	0.53(0.31)	0.75(0.20)	0.61(0.31)	-0.010	-0.02(-0.041;0.009)
*Nurse visits (N = 2*,*734)*	0.09(0.12)	0.27(0.27)	0.12(0.14)	0.25(0.24)	-0.050	**-**0.04(-0.058;-0.02)**
**Costs**						
*Total costs (N = 2*,*734)*	60.73(24.31)	59.10(26.42)	68.61(23.50)	65.03(26.05)	-1.950	-1.41(-4.452;1.619)

** indicates significant at p<0.05

### Healthcare use and costs

Greater YiL participation was not associated with any change in the risk of admissions. GP visits increased in all practices over time unrelated to the degree of participation but nurse visits in higher participation practices decreased slightly, while visits in low participation practices increased (from a low baseline). Consequently, costs of the programme–calculated as £468,530 (Table D in S1 Supporting information)—were not offset by cost savings in primary or secondary care and there was no significant difference in COPD management costs per patient between high participating and low participating practices.

### 2b. Interpreting quantitative findings

In this section we discuss three possible candidate explanations for YiL’s partial impacts (apparent improvements in guideline adherence but failure to change admissions), derived from the interviews with providers and planners. We reflect on the extent to which these data provide support for planners’ early or evolved theories or exposed flaws in them.

## YiL implementation insufficient for clinically meaningful change

In theory 2, planners believed active programme participation was necessary for providers to have the skills and tools to adopt the COPD guidelines in their clinical practice. Consistent with this theory, providers in seven out of 10 practices recalled YiL and described impacts of the educational activities, not only in terms of increasing their skills but three practitioners described it enabled them to apply their skills and three practices continued to use the YiL self-management leaflet and template:

*We’ve had our nurse do some training*, *so we’re certainly picking up more cases*. (GP, Practice 7)

*There’s so much to spirometry … when I went and did the COPD course with XXX and then on that basis I did speak to the GPs that really we need to increase our prevalence then we need to get our own spirometry*, *so we did*. (Nurse, Practice 1)

Again, consistent with planners’ evolved theory that sufficient active programme participation was needed for clinical impact, providers that recalled the programme but opportunities to participate were limited (e.g. practice staff could not attend training in work time or where they were not able to persuade practices to purchase spirometers), and reported benefits were not sustained. In addition, there were low numbers of practices with high active participation and many practices where we requested or obtained an interview did not recall the programme at all.

However, incompatible with this theory, providers and planners and documentation indicated programme participation did not always lead to higher confidence or motivation to adopt guidelines. Practices with similar levels of YiL participation expressed varying levels of confidence in managing COPD, and none of them considered YiL as the sole, or even the main determinant of better COPD management. In fact, some practices expressed considerable scepticism about the value of the feedback based on routine data, partly because of data quality concerns, but also because of suspicions about how analysis originated:

*We received your analysis of the COPD performance indicators at our practice and would like to know where the information was extracted from as it is contradictory and certainly is not equivocal [sic] to [our IT system]*. *I don’t think we have met*, *and no one here seems to know you*, *could you please let us know which company you are from?* (Practice email to YiL planners 6/2011)

Distributing feedback on performance via email in particular was not sufficient to prompt change and may actually have alienated some practices, at least initially:

*By the end of the role*, *people were desperate for the data*. *They wanted to look at it but to begin with I had a lot of meetings where they were really put out that we’d measured them without asking them*. (Planner 1, clinician)

## Planners’ and providers’ beliefs about mechanisms of change differed

In both early and evolved theories, planners sought to reduce costs through reducing emergency hospital admissions in COPD patients. Providers were generally committed to this aspiration, but considered that factors other than primary care quality were more important influences on the risk of emergency COPD admissions.

Several providers described patients’ health behaviours as most important to alter their disease trajectory to reduce the need for healthcare. This view was also echoed by one planner after the programme had finished:

Y*ou can get nurses and doctors delivering better care but until you change the behaviour of the patient all you are doing is recording data*. *Because until the patient stops smoking and actually does pulmonary rehab and understands their condition*, *they’re not going to get any better*. (Planner 1, clinician)

YiL focused on improving COPD management in primary care but providers viewed social and psychological factors as major reasons why patients attend A&E when they become unwell.

*Some people keep presenting because*, *well*, *they’re lonely and they’re unwell too*, *but they’re anxious and they need reassuring*.(Nurse, Practice 5)

Providers also described how community and hospital services influenced the likelihood of admissions:

*I have said to the respiratory nurse at XXX Hospital*, *because his*, *his argument was ‘you need to stop them coming in’ as in me*. *And I'm like ‘well you don’t have to admit them*, *you know*, *just because they walk in your door and they know what symptoms to tell you about and how to breathe*, *does not mean you have to admit them*.’ (Nurse, Practice 9)

Therefore, while providers supported the programme’s overall aim, they did not perceive their role in providing primary care in line with guidelines was key to achieving it.

## Other drivers of performance swamped programme impacts

YiL was a local programme but during 2011–2014, there were widespread efforts to improve COPD nationally[9] and regionally which raised the profile of COPD. There was evidence some practices were more strongly influenced by these drivers than YiL. For example, one entire borough declined the YiL mentorship component because “they were doing other COPD projects” and some providers that failed to recall YiL recalled other London-wide COPD interventions. Most practice staff reported the national GP contract (Quality and Outcomes Framework (QOF)) was a major influence on their time and effort in COPD:

*Wednesday morning is my QOF clinic*. *I don’t see patients*, *I spend time just looking at the registers*. (Nurse, Practice 1)

Planners sought to use these national drivers but were also aware of where their goals were not aligned. For example, the clinical lead and implementer both described how annual reviews, for which practices were remunerated by QOF, did not on their own, motivate practices to include elements recommended in guidelines:

*There was no QOF payment for doing the right thing*, *it would still be a QOF payment for doing [a review] and no one really worried too much about it*. (Planner 2, clinician)

Just as there were drivers beyond YiL towards better COPD care, there were some barriers to improving care outside YiL too. National healthcare reforms changed NHS funding and organisation and constrained stretched practice nurse capacity:

*We are very thin on the ground with practice nurses in north-east London and they’re like*, *it’s easier to get another doctor into the practice than it is to get a decent practice nurse who’s trained up*. (GP, Practice 3)

*Practices along with everywhere in the public sector*, *it’s being squeezed and squeezed*, *in the last two*, *three*, *four years*, *we have been expected to do more and more and more*. (Nurse, Practice 10)

As a result, several providers reported struggling to maintain skills and sustain the specialised activities needed to diagnose and monitor COPD patients according to guidelines.

## Discussion

### Main findings

We identified both early and evolved theories from programme planners’ ideas to evaluate a programme that sought to achieve greater implementation of COPD guidelines. Through this evaluation we illustrate how theory constructed from planners’ ideas can shape evaluation design, choice and prioritisation of questions and constructs, and also how different conclusions can result from application of different theories. Testing hypotheses based on ‘early’ theory only indicated YiL did not achieve its desired outcomes. Testing hypotheses based on ‘evolved’ theory indicated that practices with greater programme participation showed greater guideline adoption but impacts still did not extend to reducing admissions or overall costs.

### Implications of the findings

We consider the study’s implications in the context of existing research in two ways. Firstly, we describe the strengths and weakness of our approach to constructing theory retrospectively for evaluating ‘real-world’ implementation initiatives. Secondly, we discuss ways in which prospective theory articulation might and might not lead to more effective programmes.

## Learning for evaluations

We sought to derive theory through making explicit the tacit assumptions and ideas of those close to the programme in interviews after the programme and using contemporary programme documentation. This approach is advocated by Chen and others to ensure the evaluation framework is meaningful to stakeholders and measures what is important in programmes.[8]

Coryn et al’s review of 45 evaluations found that many claiming to be theory driven do not use theory “in any meaningful way” for formulating questions or informing evaluation design.[8] In contrast in this evaluation, we formulated questions based on both early and evolved constructed programme theories. It directly shaped the measures of programme participation which we used to generate comparator populations to test hypotheses based on the second theory. It also enabled us to cost the programme and its possible impacts comprehensively, a recognised limitation of previous guideline implementation studies.[22] This meant we could address some of the challenges of conducting robust evaluations of guideline implementation strategies in service settings: lack of clarity about programmes’ components and aims; evolving delivery; differential participation; without prospectively defined control groups.[12, 23]

The theory we derived however, was subject to some limitations. It was difficult to distinguish whether evolved theory captured the programme’s iteration during its lifetime or only reflected planners’ views formed after the programme had finished. Without this knowledge, it is not possible to ascertain the extent to which planners could have modified the programme in light of these realisations. Dixon-Woods et al note the limitations of constructing theory without process evaluation fieldwork data on programme evolution.[17] We contend that in many projects (like YiL) formal process evaluations are unlikely and illustrate how theory can be derived and critiqued where those close to the programme have had the courage to share documentation that open their early ideas and actions to scrutiny. Another approach would have been to construct programme theory retrospectively by applying prior theory as other evaluators have done.[8] Nilsen and others suggest middle-range theories could be used for this purpose but also recognise one theory may only provide partial understanding. Moreover, as Coryn and others warn, deriving programme theory from established theories (e.g. Theory of Planned Behaviour) may skew evaluation towards outcomes within pre-existing theory rather than what was intended by the programme.[3, 8]

There were also weaknesses in our evaluation methods that a theory-based approach could not eliminate. When the programme was implemented a control group was not included in the design (e.g., areas included in the study but not receiving the programme). Therefore it was not possible to judge the extent to which the lack of impact on admissions was due to insufficient implementation across the practices, determinants of admissions outside of YiL, or that major impacts from national system reforms/incentives swamped YiL’s impacts. This is a common problem in system-wide programme evaluations conducted in ‘real-world’ settings where experimental designs are not appropriate or feasible. To address this, we employed a difference-in-differences design, using comparator practices that had little or no participation in the YiL programme. This was still less robust than a control group because all practices had some exposure to YiL. Inaccuracies and incompleteness in routinely collected primary care data limited the conclusions we could draw from quantitative analyses. As a result, it was not possible to distinguish whether providers actually changed patient care to adopt NICE guidelines or just ensured that existing activity was better recorded. Our YiL participation index, based on registers of educational activity attendance, did not capture other important but undocumented aspects of YiL involvement (e.g. practice discussions), nor did it capture variations between staff within practices.

## Value of theory in designing interventions: Learning for planners/designers

It has been widely argued that articulation of theory should lead to more effective programmes.[4, 13] There are frameworks and process models developed for such articulation[4] but programme designers can be reluctant to develop theory and may struggle to see its value.[7] Moreover, there is little empirical evidence from programmes developed outside research that theory does lead to better programmes.

Our study did not set out to test whether prospective theory articulation could improve programmes’ chances of success because it was a retrospective study. However, it suggests ways in which prospective theory might or might not have improved this programme that may be generalizable to other initiatives.

On one hand, retrospective theory construction made clear that the programme started with a lack of theory, particularly concerning mechanisms of change. In particular it highlighted an important flaw in early theory concerning programme implementation (i.e. unanticipated problems with using routine data for feedback to drive change). Planners recognised the flaw soon after the programme’s start. They changed implementation tactics (improving data quality, extraction and feedback mechanisms) and altered their underlying ideas about how change would occur (recognising the need for training and tools to enable practices to improve care). In this case, prospective theory articulation could have forced planners to make explicit the mechanisms by which they thought change could occur. There is a wealth of existing evidence on data and feedback that could have informed this aspect of the programme.[24, 25] Moreover, existing implementation frameworks or process models could prompt programme planners or designers to break down the steps involved in implementing programmes[7] and consider factors such as organisational readiness for change[26] to anticipate and address implementation problems before a programme starts.

On the other hand, formally setting out theory at the beginning of YiL–even using existing implementation models—was unlikely to have altered planners’ aspiration to reduce emergency hospital admissions. This aspiration looked reasonable at the time YiL was instigated; it was assumed in national guidelines and COPD strategy documents that reduced admissions through better primary care was ‘likely’.[27, 21] This assumption—based on professional consensus not empirical data–has since been questioned, in that few initiatives have succeeded in reducing emergency admissions.[28–33]

NICE COPD guidelines are not unusual in lacking underpinning empirical evidence. Guidelines are based on robust empirical evidence of patient benefit where possible, but they are still formed where empirical evidence is lacking. Even where underpinning evidence is available, an evidence base drawn from one population may not be applicable to a different target patient population.[34, 35] Applying implementation theories or frameworks, which generally do not go beyond guideline implementation to consider health outcomes, would not prompt planners to challenge the feasibility of achieving health outcomes either.

Renger et al speculate that programme designers may also fear “making things explicit” through articulating programme theory.[6] For YiL, reducing emergency hospital admissions was central to several planners’ motivation to initiate the programme, above and beyond guideline adherence. This was (and remains) an NHS policy priority.[36, 37]. Therefore, planners could have been reluctant to challenge how and whether it was possible to achieve it. More broadly, our evaluation suggests that loose, informal theory may have kept several planners with different ideas involved in YiL while they all learnt more about the context and implementation of the programme. Confronting a lack of consensus early in a programme where there are many unknowns could alienate some planners and put a programme in jeopardy.

## Conclusions

In the UK, it is expected that local services will promote adherence to national guidelines but programmes to implement such guidance often have limited success. Moreover evaluations of programmes conducted in real-world settings are often not captured in evidence reviews. Our theory-based evaluation of a real-world programme indicates that reconstructing theories used by programmes planners can provide the basis for more robust evaluation.

It is also widely argued that applying theory would lead to better programmes. Our evaluation supports the call for planners to prospectively articulate theory, drawing on existing evidence of successful implementation, theories and models to strengthen implementation of guideline adoption efforts. However, planners’ motivations to initiate such programmes (and providers to implement guidelines) depend on the belief that implementation will improve health outcomes. Where this supporting evidence is weak or uncertain, prospectively articulating or using existing theory may not lead to more successful programmes.

## Supporting information

S1 Supporting information Additional description of quantitative methods and additional results tables for theories 1 and 2. (DOCX)
