# Supplementary material for: The value of theory in programmes to implement clinical guidelines: Insights from a retrospective mixed-methods evaluation of a programme to increase adherence to national guidelines for chronic disease in primary care
Source: PLoS One. 2017 Mar 22;12(3):e0174086. doi: 10.1371/journal.pone.0174086 (PMC5362095; doi:10.1371/journal.pone.0174086)
Supplement: S1 Supporting information — (DOCX) [file pone.0174086.s001.docx]

# **Article title: The value of theory in programmes to implement clinical guidelines: Insights from a retrospective mixed-methods evaluation of a programme to increase adherence to national guidelines for chronic disease in primary care**

**S1. Supporting Information**

To complement the main paper we report here more detail on the datasets and analyses for Theories 1 and 2 and results from alternative analyses performed.

**Theory 1 (Early)**

**METHODS**

**Definition of outcome**

To be considered as a COPD diagnosis either of the following two definitions needs to be met, based on a recorded primary diagnosis in HES containing the International Classification of Disease 10^th^ Edition (ICD-10) codes.^1,2^

- 1. First diagnoses contains: 'J41', 'J42', 'J43', 'J44', 'J47'
  2. or First diagnosis code contains 'J20' and second diagnosis code contains 'J41', 'J42', 'J43', 'J44', 'J47’

**Definition of population**

To establish the rates the following population definitions were used:

1. Time period:
   1. All COPD emergency hospital admissions (EHA) in the period 1 January 2004 to 31 December 2013.
2. Outer North East London boroughs:
   1. Barking and Dagenham
   2. Havering
   3. Redbridge
   4. Waltham Forest
3. Denominator (population at risk):
   1. The age and gender stratified population of England as provided by the ONS at the level of the lower super output authority (LSOA) for the years 2004 to 2012.
   2. LSOAs were mapped to local authorities to allow aggregation at the LA level for analysis
   3. The 2012 population was used as the denominator for 2013 emergency admissions of COPD to allow for the most recent cases
4. Numerator (patients who had a diagnosis of COPD)
5. All patients who had an inpatient non-elective (emergency) admission and met the COPD diagnosis criteria defined above during the defined time period
6. Patient stayed for at least one night
7. Creating the analysis population
8. The denominator and numerator populations are stratified by month of analysis, age, gender, deprivation decile and LA
9. The two populations are then joined based on matching each level of the strata

Comparator boroughs were selected using ONS corresponding local authorities (see Table A).

**Table A. ONS comparable local authorities**

|  | Closest comparable borough as per ONS^[[1]](#footnote-1)^ | | | | |
| --- | --- | --- | --- | --- | --- |
| ONEL Borough | First | Second | Third | Fourth | Fifth |
| Barking and Dagenham (00AB) | ~~Greenwich (00AL)~~ | Enfield (00AK) | Birmingham (00CN) | Sandwell (00CS) | ~~Bexley (00AD)~~ |
| Havering (00AR) | ~~Bexley~~  (~~00AD)~~ | Epping Forrest (22UH) | Basildon (22UB) | Rochford (22UL) | Broxbourne (26UB) |
| Redbridge (00BC) | Harrow (00AO) | Ealing  (00AJ) | Hounslow (00AT) | Barnet (00AC) | Luton  (00KA) |
| Waltham Forrest (00BH) | Croydon (00AH) | ~~Greenwich (00AL)~~ | Enfield (00AK) | Haringey (00AP) | Merton (00BA) |

**Note:** Due to non-reporting by South London Healthcare Trust in the period April 2013 to September 2013 Greenwich, Bexley and Bromley local authorities have been excluded from the analysis.

**Analyses**

1. Descriptive:
   1. National crude rates of COPD emergency admissions
   2. Sex, age and deprivation standardised rates for Outer North East London (ONEL) boroughs
   3. Sex, age and deprivation standardised for ONEL and Non-ONEL local authorities (LA)
   4. Sex, age and deprivation standardised for ONEL and a selection of ONEL comparator LAs as defined by the Office of National Statistics (ONS)
2. Inferential
   1. To evaluate the differences between the ONEL boroughs and ONEL comparator local authorities rates of emergency admissions in the before, during and after periods of the intervention implementation. The analysis will be adjusted for age group, gender, deprivation decile and month (seasonality).

Crude COPD emergency admissions rates per month per 1,000 populations were calculated for each calendar month from January 2004 to 31 December 2013 to show the overall trend, predating YiL. Age, gender and deprivation directly standardised rates were calculated for the ONEL boroughs and ONEL comparator boroughs for each calendar month.

To calculate COPD emergency admissions rates between the ONEL and ONEL comparator LAs a Poisson model was constructed with the number of emergency admissions as the outcome variable and the log of the population included as an offset variable. Age was included as a categorical variable in five year bands, gender was included as a binary variable, and deprivation decile was included as a categorical variable with ten levels. A twelve level categorical variable for calendar month was used to account for seasonality.

The exposure variable was defined as a binary variable taking the value one if the population group belonged to an ONEL borough and the value zero if the group belonged to an ONEL comparator LA.

The analysis was restricted to the period from 1 January 2009 to 31 December 2013 to reduce the impact of trends from earlier time periods being projected into the more recent periods. This was further refined by dividing the analysis period into three categories:

1. Before intervention implementation period from 1 January 2009 until 30 September 2011.
2. During intervention implementation period from 1 October 2011 until 31 December 2012
3. After intervention implementation period from 1 January 2013 until 31 December 2013

The primary analysis of the impact of the intervention is the interaction of the exposure variable (ONEL borough yes/no) with the analysis period variable, after adjustment for age, gender, deprivation and month (seasonality). To ensure p-values are valid empirical standard errors are calculated using General Estimating Equations (GEE) with an independent working correlation matrix to account for the correlation of outcomes within a LA.

**RESULTS**

To complement the analysis presented in the main paper, we report here:

- national crude rates of EHA 2004-2013 (Figure A)
- standardised rates 2009-2013: ONEL vs comparators; ONEL vs national (remaining boroughs) (Figure B)
- rate ratios: ONEL vs comparators in each time period and between time periods (Tables B and C)

**Figure A: Crude rates of COPD emergency admissions in ONEL boroughs January 2004 to December 2013**


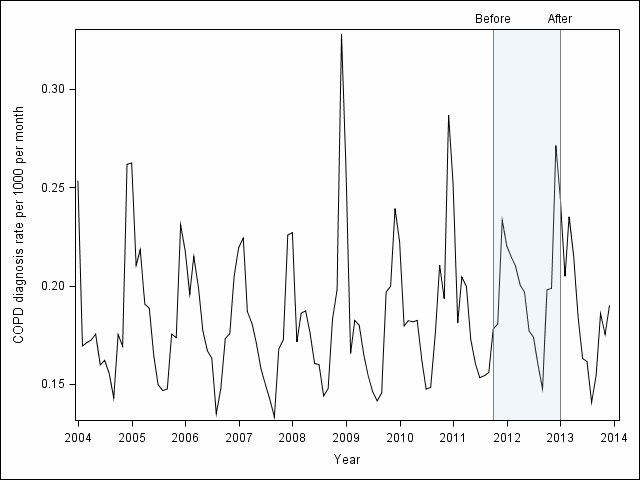


**Figure B: Age, gender and deprivation standardised rates of COPD emergency admissions: ONEL versus comparators v national: 1 January 2009 to 31 December 2013**


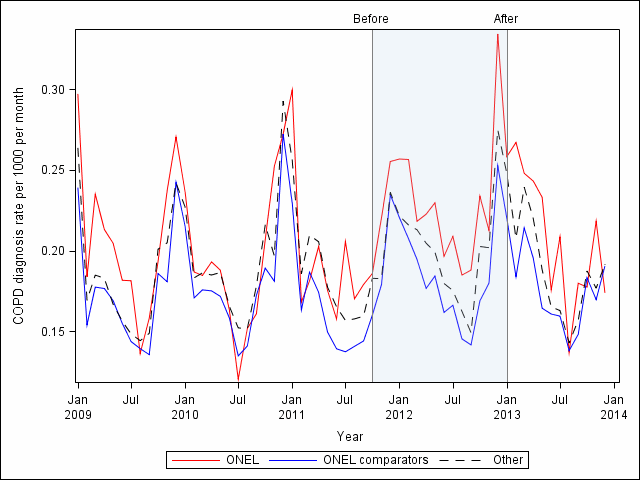


**Table B: Rate ratios and 95% confidence intervals for ONEL versus ONEL comparators in each time period**

| **Time period** | **Exposure** | **Rate ratio^[[2]](#footnote-2)^** | **95% CI** |
| --- | --- | --- | --- |
| Before |  |  |  |
|  | Comparator | 1.000 |  |
|  | ONEL | 1.135 | (0.796, 1.619) |
| During |  |  |  |
|  | Comparator | 1.000 |  |
|  | ONEL | 1.223 | (0.839, 1.783) |
| After |  |  |  |
|  | Comparator | 1.000 |  |
|  | ONEL | 1.183 | (0.810, 1.727) |

**Table C: Rate ratios and 95% confidence intervals for each time period for ONEL and ONEL comparators**

| **Exposure** | **Time period** | **Rate ratio^[[3]](#footnote-3)^** | **95% CI** |
| --- | --- | --- | --- |
| ONEL |  |  |  |
|  | Before | 1.000 |  |
|  | During | 1.100 | (1.029, 1.175) |
|  | After | 1.049 | (0.956, 1.151) |
| ONEL comparators |  |  |  |
|  | Before | 1.000 |  |
|  | During | 1.021 | (1.000, 1.042) |
|  | After | 1.007 | (0.966, 1.050) |

#### **Theory 2 (‘evolved’)**

**METHODS**

**Sample**

We included patients from the month of first diagnosis of COPD (if that occurred prior to 2010 they were included at start of study period) and dropped them if they left the practice or died. The final dataset consisted of repeated (i.e. monthly) observations for each patient (range 1 – 46 months). From an initial sample of 577,979 patient-month observations, we excluded patients with incomplete data on socio-demographic variables (N= 58,141 patient-months, 10%) and missing data on GP and nurse consultations (N= 6,610 patient-months, 1.3%). For all other COPD-related measures data were extracted from read codes referring to single consultations. Values missing for months without a consultation were carried forward from the previous consultation where the measure was recorded. If no data were ever available on a specific measure, the variable was coded as ’measurement not taken/recorded’ and obvious data errors (e.g. patients with a date of diagnosis dating 1900) were recoded as missing and excluded from analyses.

**Variables**

***Exposures***

We employed Programme time periods (before, during, after, as previously defined) as our main exposure variable and practices’ active participation in YiL.

To examine the effect of active participation in YiL, we created a variable to quantify participation of each GP practice. Using attendance lists for all YiL educational activities and records of whether practices uploaded the COPD template, we computed how many interventions each practice undertook whether they undertook them fully or partially, and when they did so. Level of participation was defined for each practice as that achieved by the end of the follow-up period in order to have distinctly comparable groups throughout the period. This allowed us to create a dataset where for every practice we assigned a score describing total uptake of YiL interventions ranging from 0 (no recorded participation) to 4 (uptake of all YiL interventions) on a month-by-month basis.

***Outcomes***

**Diagnosis and management of COPD in primary care**

We defined three binary (yes/no) proximal outcomes indicating whether the patient had ever received: (i) post-bronchodilator spirometry to confirm diagnosis; (ii) a self-management plan; and (iii) a referral for pulmonary rehabilitation (in patients with severe COPD defined as Grade 3-5 on the MRC scale). We also included a binary variable indicating prescription of inhaled corticosteroids (ICS) in isolation of other COPD medications in each month.

**Healthcare use and cost**

We created two count variables indicating number of nurses and GP consultations in each calendar month (primary care outcomes), and a binary (yes/no) variable indicating whether, in each calendar month, the patient had any emergency hospital admissions (EHA) with a primary diagnosis of COPD (secondary care outcome), defined using the same ICD 10 codes as used in Theory 1.

We collected data on two cost components using an NHS perspective: (i) the cost of planning, coordinating, and delivering YiL; (ii) COPD-related healthcare costs for each COPD patient.

For the first component, YiL planners supplied: data on the resources used for the preparation and delivery of the masterclasses, spirometry training, nurse mentorship and the dashboard. These included venue hire, staff time arranging, co-ordinating and delivering interventions and NHS staff costs of those attending masterclasses and spirometry training. We estimated the costs of: ongoing coordination, communication and strategic input from the YiL steering group using YiL documentation; and of developing the informatics necessary to generate credible and regular benchmarking data on COPD processes of care using data supplied by YiL planners.

For the second component, we derived two COPD-related cost measures: total primary care costs; and, costs of EHA using unit costs derived from the NHS Reference Costs.^3^ For primary care costs, we included GP and practice nurse consultations at a cost of £45 and £13 per consultation, respectively, plus the cost of the following medications: short-acting beta agonist (SABA, £2.29 per month), inhaled corticosteroid (ICS, £3.05), short-acting muscarinic antagonist (SAMA, £2.46), LABA (£31.72), long-acting muscarinic antagonist (LAMA, £35.22), LABA+ICS (£41.19) using estimates produced by NICE.^4^ We estimated the cost of an EHA as £1,190.

***Other variables***

We also included a number of time variant and invariant patient-level variables in our analyses.

Time variant variables were: smoking (never smoker, current smoker, ex-smoker); COPD severity according to the MRC severity scale (range 1- 5 from less to more severe) including a separate category if measurement had never been taken; whether the patient had an EHA in the previous 12 months.

The time-invariant variables were: gender (male, female); Index of Multiple Deprivation quintiles based on area of residence, ethnicity (White, Black, Asian, Mixed, Other, not stated/recorded), age at inclusion in COPD register, and comorbidity (asthma, chronic kidney disease, heart failure and diabetes ; yes/no). Additionally, we created two indicator variables to flag participants with potentially erroneous data: recorded age of diagnosis (<40 years of age; 1.56% patients) and smoking status (two conflicting read-codes on same date; 1.55%).

**Alternative analyses performed**

We evaluated whether YiL had an impact on improving the set of outcomes previously defined using a Difference-in-differences (DiD) regression analysis to compare changes in practices by participation and over time. In the main paper we report difference in difference analyses comparing ‘before and during’ vs ‘after’ and practice participation: low (score of 0-1) vs higher (score of 2-4). To examine whether our findings were sensitive to the time periods compared or the degree of participation chosen, we analysed the data in a number of different ways.

**RESULTS**

We report here:

- patient-level characteristics of the dataset before, during and after YiL (Table D)
- breakdown of the estimated costs of the programme (Table E)
- alternative difference in difference analyses (Tables F,G and H):
  - Comparing ‘before’ vs ‘during and after’ and practice participation: low (score of 0-1) vs higher (score of 2-4).
  - Comparing ‘before’ vs ‘during and after’ and practice participation: lower (score of 0-3) vs maximum (score of 4).
  - Comparing ‘before and during’ vs ‘after’ and practice participation: lower (score of 0-3) vs maximum (score of 4).

**Table D. Programme cost: breakdown by component**

| **Cost component** | **Amount (£)** |
| --- | --- |
| Co-ordination including additional facilities and administration costs | 190,302 |
| Masterclasses and Spirometry Training | 83,533 |
| Nurse Mentorship | 47,409 |
| ONEL template | 37,416 |
| Self-management plans | 29,870 |
| Health Analytics/Dashboards | 80,000 |
| Total | 468,530 |

Tables S2.3-5 show there was some difference in the significance of the findings depending on which analyses were undertaken but the main findings - changes in adherence but no change in admissions - remained constant across all the analysis.

Table E. Distribution of patient-level characteristics before, during and after YiL (N= 513,228 patient months)

|  | **Before** | **During** | **After** |
| --- | --- | --- | --- |
| **Age at entry to the COPD register (mean (SD)** | 68.80(12.03) | 67.64(12.39) | 66.88(12.41) |
| **Gender** | **n(%)** | **n(%)** | **n(%)** |
| *Male* | 91,790(49.15%) | 84,585(48.70%) | 73,527(48.12%) |
| *Female* | 95,962(50.85%) | 89,094(51.30%) | 79,270(51.88%) |
| **Ethnicity** |  |  |  |
| *White* | 77,923(41.73%) | 71,254(41.03%) | 62,681(41.02%) |
| *Black* | 3.090(1.65%) | 3,129(1.80%) | 3,034(1.99%) |
| *Asian* | 10,257(5.49%) | 10,623(36.09%) | 9,787(6.41%) |
| *Mixed* | 67,477(36.13%) | 62,673(36.09%) | 53,998(35.34%) |
| *Other* | 26,853(14.38%) | 24,963(14.37%) | 22,367(14,64%) |
| *Not stated/recorded* | 1,152(0.62%) | 1,037(0.60%) | 930(0.61%) |
| **IMD Quintile** |  |  |  |
| *1 (least deprived)* | 38,244(20.48%) | 34,952(20.12%) | 30,756(20.13%) |
| *2* | 36,857(19.74%) | 33,829(19.48%) | 29,926(19.59%) |
| *3* | 38,025(20.36%) | 35,312(20.335) | 30,727(20.11%) |
| *4* | 36,359(19.47%) | 34,475(19.85%) | 30,747(20.12%) |
| *5(most deprived)* | 37,267(19.96%) | 35,111(20.22%) | 30,641(20.05%) |
| **Smoking** |  |  |  |
| *Never smoker* | 32,493(17.40%) | 28,975(16.68%) | 25,649(16.79%) |
| *Current Smoker* | 60,007(32.13%) | 56,865(32.74%) | 49,559(32.43%) |
| *Ex-smoker* | 94,252(50.47%) | 87,839(50.58%) | 77,589(50.78%) |
| **COPD severity (MRC grade)** |  |  |  |
| 1 (least severe) | 33,175(17.76%) | 30,482(17.55%) | 28,252(18.49%) |
| 2 | 60,076(32.17%) | 55,141(31.75%) | 49,977(32.71%) |
| 3 | 47,359(25.36%) | 45,459(26.17%) | 38,950(25.49%) |
| 4 | 29,095(15.58%) | 26,843(15.46%) | 22,571(14.77%) |
| 5(Most severe) | 8,374(4.48%) | 7,672(4.42%) | 5,749(3.76%) |
| Not measured | 8,673(4.64%) | 8,082(4.65%) | 7,298(4.78%) |
| **Recorded comorbidities** |  |  |  |
| Asthma | 55,520(29.73%) | 51,552(29.68%) | 45,283(29.64%) |
| Diabetes | 35,258(18.88%) | 32,213(18.55) | 27,724(18.14%) |
| CKD | 33,621(18%) | 28,837(16.60%) | 23,652(15.48%) |
| Heart Failure | 15,884(8.51%) | 13,195(7.60%) | 10,375(6.79%) |
| **Borough** |  |  |  |
| *1* | 44,101(23.61%) | 42,491(24.47%) | 37,917(24.82%) |
| *2* | 59,582(31.90%) | 54,886(31.60%) | 47,628(31.17%) |
| *3* | 40,469(21.67%) | 36,557(21.05%) | 32,736(21.42%) |
| *4* | 42,600(22.81%) | 39,745(22.88%) | 34,516(22.59%) |
|  |  |  |  |
| **Total number of patient months** | 186,752 | 173,679 | 152,797 |

**Table F. Difference in differences results comparing ‘before’ vs ‘during and after’ periods of YiL and practice participation: low vs higher.**

|  | **Before + During** | | **After** | | **Unadjusted difference in differences** | **Difference in differences, coeff (95%CI)** |
| --- | --- | --- | --- | --- | --- | --- |
| **Outcome**  **‘before and during’ vs ‘after’** | **Part. 0-1,**  **Mean (SD)**  **(N=605)** | **Part. 2-4,**  **Mean (SD)**  **(N=1401)** | **Part. 0-1,**  **Mean (SD)**  **(N=605)** | **Part. 2-4,**  **Mean (SD)**  **(N=1401)** |  |  |
| *Spirometry (N=2,734)* | 0.61(0.17) | 0.62(0.17) | 0.60(0.17) | 0.60(0.17) | -0.010 | -0.012(-0.025;0.009) |
| *PBD spirometry (N=2,734)* | 0.08(0.13) | 0.14(0.18) | 0.23(0.20) | 0.37(0.23) | 0.080 | **0.08(0.059;0.093)**** |
| *Self-management plan(N=2,734)* | 0.05(0.12) | 0.09(0.18) | 0.16(0.22) | 0.26(0.24) | 0.060 | **0.06(0.041;0.081)**** |
| *Pulmonary Rehab (ever) (N=2,728)* | 0.01(0.03) | 0.04(0.10) | 0.03(0.07) | 0.09(0.15) | 0.030 | **0.03(0.024;0.045)**** |
| *ICS mild cases (N=2,718)* | 0.003(0.01) | 0.004(0.01) | 0.0008(0.004) | 0.0007(0.003) | -0.001 | -0.0001(-0.002;0.001) |
| *ICS severe cases (N=2,728)* | 0.002(0.01) | 0.003(0.01) | 0.001(0.005) | 0.001(0.005) | -0.001 | -0.0002(-0.002;0.002) |
| *GP visits (N=2,734)* | 0.69(0.24) | 0.56(0.32) | 0.77(0.18) | 0.63(0.29) | -0.010 | -0.02(-0.04;0.01) |
| *Nurse visits (N=2,734)* | 0.11(0.14) | 0.27(0.26) | 0.11(0.13) | 0.22(0.23) | -0.050 | **-0.05(-0.069;-0.022)**** |
| *Admissions (N=2,734)* | 0.008(0.013) | 0.009(0.013) | 0.009(0.12) | 0.008(0.011) | -0.002 | -0.001(-0.003;0.001) |
| *Total costs (N=2,734)* | 63.82(24.88) | 61.94(26.53) | 69.96(21.33) | 64.60(25.76) | -3.48 | **-2.93(-6.28;0.43)**** |

**Table G: Difference in differences results comparing ‘before and during’ vs ‘after’ periods of YiL and lower vs maximum practice participation**

|  | **Before + During intervention** | | **After intervention** | | **Unadjusted difference in differences** | **Difference in differences, coeff (95%CI)** |
| --- | --- | --- | --- | --- | --- | --- |
| **Outcome** | **Part. 0-3,**  **Mean (SD)** | **Part. 4,**  **Mean (SD)** | **Part. 0-3,**  **Mean (SD)** | **Part. 4,**  **Mean (SD)** |  |  |
| Spirometry (N=2,734) | 0.62(0.17) | 0.62(0.16) | 0.60(0.17) | 0.58(0.18) | -0.02 | -.03(-0.07;0.1) |
| PBD spirometry (N=2,734) | 0.12(0.18) | 0.10(0.14) | 0.32(0.23) | 0.39(0.22) | 0.09 | **0.08(0.05;0.11)**** |
| Self-management plan(N=2,734) | 0.07(0.16) | 0.12(0.22) | 0.22(0.24) | 0.37(0.22) | 0.1 | **0.11(0.07-0.14)**** |
| Pulmonary Rehab (ever) (N=2,728) | 0.03(0.09) | 0.04(0.05) | 0.07(0.13) | 0.14(0.08) | 0.06 | **0.05(0.03;0.07)**** |
| ICS mild cases (N=2,718) | 0.004(0.01) | 0.002(0.005) | 0.0007(0.003) | 0.0009(0.004) | 0.0022 | 0.003(0.0001;0.006) |
| ICS severe cases (N=2,728) | 0.003(0.01) | 0.003(0.009) | 0.001(0.005) | 0.0008(0.004) | -0.002 | 0.0002(-0.003;0.004) |
| GP visits (N=2,734) | 0.61(0.30) | 0.51(0.26) | 0.68(0.27) | 0.52(0.28) | -0.06 | **-0.06(-0.11;-0.01)*** |
| Nurse visits (N=2,734) | 0.22(0.24) | 0.30(0.27) | 0.18(0.21) | 0.24(0.28) | -0.02 | -0.03(-0.07;0.02) |
| Admissions (N=2,734) | 0.009(0.01) | 0.0086(0.01) | 0.009(0.01) | 0.0083(0.01) | -0.0003 | 0.0004(-0.003;0.004) |
| Total costs (N=2,734) | 62.7(26.2) | 59,8(23.7) |  | 58.4(23.08) | -5.53 | ***-5.07(-11.06;0.92)*** |

**Table H: Difference in differences results comparing ‘before’ vs ‘during and after’ periods of YiL and lower vs maximum practice participation**

|  | **Before intervention** | | **During + After**  **intervention** | | **Unadjusted difference in differences** | **Difference in differences, coeff (95%CI)** |
| --- | --- | --- | --- | --- | --- | --- |
| **Outcome** | **Part. 0-3,**  **Mean (SD)** | **Part. 4,**  **Mean (SD)** | **Part. 0-3,**  **Mean (SD)** | **Part. 4,**  **Mean (SD)** |  |  |
| Spirometry (N=2,734) | 0.63(0.17) | 0.64(0.17) | 0.60(0.17) | 0.59(0.16) | -0.02 | -0.01(-0.05;0.02) |
| PBD spirometry (N=2,734) | 0.07(0.14) | 0.04(0.08) | 0.25(0.22) | 0.27(0.21) | 0.05 | **0.05(0.03;0.08)**** |
| Self-management plan(N=2,734) | 0.03(0.11) | 0.04(0.15) | 0.16(0.22) | 0.28(0.25) | 0.11 | **0.12(0.08;0.15)**** |
| Pulmonary Rehab (ever) (N=2,728) | 0.02(0.07) | 0.02(0.03) | 0.06(0.12) | 0.10(0.08) | 0.04 | **0.04(0.03;0.06)** |
| ICS mild cases (N=2,718) | 0.005(0.01) | 0.003(0.006) | 0.002(0.007) | 0.0008(0.003) | 0.0008 | 0.001(-0.001;0.004) |
| ICS severe cases (N=2,728) | 0.003(0.01) | 0.004(0.01) | 0.002(0.01) | 0.0008(0.003) | -0.0022 | -0.002(-0.005;0.002) |
| GP visits (N=2,734) | 0.58(0.30) | 0.47(0.26) | 0.66(0.28) | 0.54(0.28) | -0.01 | -0.02(-0.06;0.02) |
| Nurse visits (N=2,734) | 0.21(0.25) | 0.31(0.27) | 0.21(0.22) | 0.27(0.28) | -0.04 | ***-0.04(-0.07;0.002)*** |
| Admissions (N=2,734) | 0.008(0.012) | 0.009(0.01) | 0.009(0.01) | 0.009(0.01) | -0.001 | 0.003(-0.003;0.004) |
| Total costs (N=2,734) |  |  |  |  |  | -2.03(-7.44;3.37) |

**REFERENCES**

1. Organisation WH. International statistical classification of diseases and related health problems. - 10th revision, edition 2010. In: WHO, ed. 2010 ed, 2011.

2. Bardsley M, Blunt I, Davies S, et al. Is secondary preventive care improving? Observational study of 10-year trends in emergency admissions for conditions amenable to ambulatory care. BMJ open 2013;**3**(1).

3. Curtis L. Unit Costs of Health and Social Care 2011. Secondary Unit Costs of Health and Social Care 2011. 2011. [www.pssru.ac.uk/project-pages/unit-costs/2011/index.php](http://www.pssru.ac.uk/project-pages/unit-costs/2011/index.php).

4. NICE. Chronic obstructive pulmonary disease Costing report: Implementing NICE guidance, 2011.

1. http://www.ons.gov.uk/ons/guide-method/geography/products/area-classifications/ns-area-classifications/index/corresponding-authorities/local-authorities/corresponding-las.xls [↑](#footnote-ref-1)
2. Adjusted for age, gender, deprivation decile and month (seasonality) [↑](#footnote-ref-2)
3. Adjusted for age, gender, deprivation decile and month (seasonality) [↑](#footnote-ref-3)
